# Australian Fish Stretching Their Legs

**DOI:** 10.1371/journal.pbio.1001167

**Published:** 2011-10-04

**Authors:** Erik Vance

**Affiliations:** Freelance Science Writer, Mexico City, Mexico

**Figure:**
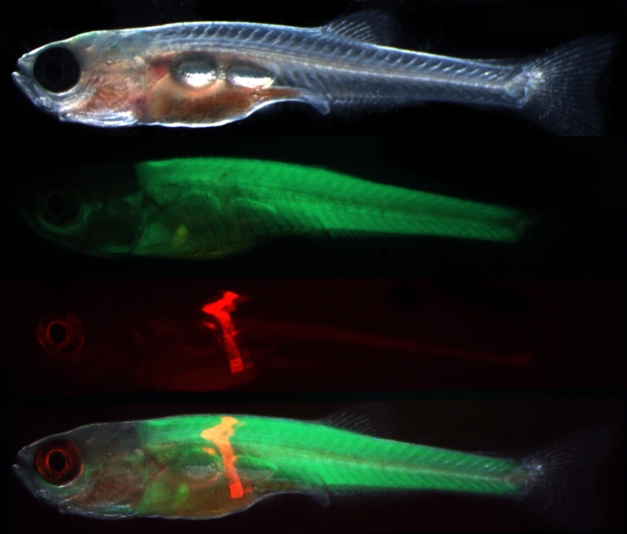
Somite transplantation in *D. rerio*. The donor somite from a transgenic fish with red fluorescent muscle is transplanted into a host that has green fluorescent muscle to reveal the myotomal origins of the pelvic fin musculature. Image credit: Nicholas J. Cole.

Anyone who has even the dimmest conception of evolution has seen the iconic image of a trail of creatures crawling out of the ocean, growing hair, gradually standing erect, and eventually becoming Man. It seems easy enough, but each stage of that 3-billion-year journey was a labored and complicated process.

This is no truer than with that crucial step from the ocean to the land. Since the earliest days of evolutionary theory, scientists have longed to understand just how that process occurred and what the intermediate steps looked like. They have compiled a detailed picture of the process of how the bones evolved to support the animal's weight on land, but very little is known about the other half of the story—how the muscles adapted and changed.

Little is known about how muscle development facilitated the transfer from ocean to land because muscles, unlike bone, haven't been preserved in the fossil record. However, it's possible to glimpse that process as an embryo grows, presuming you know where to look. That's where a group of Australian scientists come in. Over an exhausting 6 years, a team led by Peter Currie at Monash University and Nicolas Cole at the University of Sydney created a detailed picture of how the muscles in the pelvic bones of fish evolved in order to prepare them to crawl onto land.

Their paper, in this week's *PLoS Biology*, details their work in two distinct parts. The first involved a novel laboratory technique that has previously only been used with chickens and mice. The team bred two strains of zebrafish (Cyprinidae of the order Cypriniformes), a common laboratory animal. One strain was engineered to have muscle tissue that, as an embryo, glowed a soft green color and the other a soft red. Then they transplanted muscle tissue from the pelvic region from the red embryo to a “green” fish before the muscles in the pelvic fins had fully developed (this in itself is an accomplishment since the authors maintain that in no other species can you transplant genetically marked tissue between embryos). The team chose the pelvic region despite the fact that it develops late, making it hard to study. For fish, it's not the pelvis, but the front pectoral fins (as well as the tail) that dominate locomotion. But as animals moved from the ocean to land, that dominance shifted and the back legs and pelvic muscles became far more important to locomotion. This change, they theorized, would be reflected in embryonic pelvic muscles.

Sure enough, once the transplanted red muscle tissue established itself in the “green“ fish, it began to develop normally. The scientists then tracked the muscle growth, which revealed several groups of tissues—just a few cells in size—that migrated toward the pelvic region and seemed to be an early version of the muscles that made the move to land possible.

But the team was far from done. At the same time, they began collecting embryos from the bamboo shark, *Chiloscyllium punctatum*, the bizarre-looking chimera (*Callorhinchus milii*), the North American paddlefish (*Polyodon spathula*), and the Australian lungfish (*Neoceratodus forsteri*) to add to what they had learned about the zebrafish. These species were not chosen at random, but rather represented a wide swath of fish, from the ancient cartilage-skeleton sharks and chimeras to the fish with bony skeletons such as paddlefish and lungfish, the latter of which is among the closest fish to those that crawled from the ocean.

It wasn't easy, since many of the embryos had to be collected at a specific time of year and species like the chimera had never been kept in captivity at that stage of life. The work involved numerous trips to ocean sites around Australia and close collaboration with labs overseas with access to embryos. The hard work paid off. Along with what they had discovered in zebrafish, the researchers managed to chart the development of the muscle groups in fish that likely enabled them to convert to the tetrapod body type, which was better able to support its weight and move around on land. They confirmed that pelvic fin muscle of cartilaginous fishes develops by myotomal extension, that is, the muscles are ultimately extensions of the body wall muscles. However, they found in the bony fishes that the pelvic muscles develop via an initial “primitive mode” of myotomal extension towards the base of the fin where they drop epithelial cells. The cells subsequently undergo a transformation into migratory mesenchymal cells—they switch on a set of genes that represents an early version of that in limb muscles in terrestrial animals—and then spread into the rest of the fin.

Finding a developmental “transitional” process is rare in evolution research without a time machine to take one back to inspect a long-extinct transitional species. It demonstrates that the processes that were important for locomotion on land were already in place in an early form in bony fish.

**Cole NJ, Hall TE, Don EK, Berger S, Boisvert CA, et al. (2011) Development and Evolution of the Muscles of the Pelvic Fin. doi:10.1371/journal.pbio.1001168**

